# Changes in Biogenic Amines of Two Table Grapes (cv. Bronx Seedless and Italia) during Berry Development and Ripening

**DOI:** 10.3390/plants11212845

**Published:** 2022-10-26

**Authors:** Melek Incesu, Sinem Karakus, Hanifeh Seyed Hajizadeh, Fadime Ates, Metin Turan, Milan Skalicky, Ozkan Kaya

**Affiliations:** 1 Department of Food Engineering, Faculty of Agriculture, Ataturk University, Erzurum 25100, Turkey; 2 Çölemerik Vocational School, Hakkari University, Hakkari 30000, Turkey; 3 Department of Biology, Faculty of Science and Art, Erzincan Binali Yıldırım University, Erzincan 24002, Turkey; 4 Department of Horticulture, Faculty of Agriculture, University of Maragheh, Maragheh 55136-553, Iran; 5 Manisa Viticulture Research Institute, Republic of Turkey Ministry of Agriculture and Forestry, Manisa 45125, Turkey; 6 Department of Genetics and Bioengineering, Faculty of Engineering, Yeditepe University, Istanbul 34755, Turkey; 7 Department of Botany and Plant Physiology, Faculty of Agrobiology, Food and Natural Resources, Czech University of Life Sciences Prague, Kamycka 129, 16500 Prague, Czech Republic; 8 Erzincan Horticultural Research Institute, Republic of Turkey Ministry of Agriculture and Forestry, Erzincan 24060, Turkey

**Keywords:** grape, histamine, putrescine, agmatine, dopamine, spermine

## Abstract

Bronx Seedless and Italia (*Vitis vinifera* L.) are a variety preferred by consumers owing to their exciting flavour and widely cultivated in Aegean Region in Turkey. The aim was to identify the biogenic amines of these table grapes during berry ripeness. The biogenic amines were analyzed by HPLC in six different berry phenological stages. Italia grapes presented lower biogenic amine content than Bronx Seedless table grapes. The concentration of most of the biogenic amines analyzed linearly raised from the beginning of berry touch to when berries ripen for harvest stages. The most common biogenic amines in grape varieties were putrescine, followed by histamine, agmatine, and tyramine. There was also a positive correlation between all biogenic amines of the two grape varieties. The weakest correlation was found between spermine and cadaverine, whereas the strongest correlation was found among dopamine, trimethylamine, norepinephrine, tyramine, and histamine amines. The present study is the first report of a synthesis study regarding the effect of B.A.s on quality characteristics throughout berry ripeness in grape varieties containing foxy and muscat tastes. The concentration and composition of biogenic amines identified for both varieties might provide helpful information regarding human health and the vintage.

## 1. Introduction

Global grape production reaches roughly 75 million tons annually, making grapes (*Vitis* sp.) one of the most cultivated fruit crops worldwide [1,2,3]. It has significant amounts of organic compounds, minerals, and carbohydrates, as well as its production, which increases day by day due to its derivatives, such as raisins, wine, fruit juice, jam, and flour consumption, which are known to be beneficial for human health [4]. The monitoring of these organic compounds is very important to producers because efficient quality control is needed, and their profile could influence the acceptability of consumers. It has been, indeed, noted that grape derivatives have a positive role in the prevention of various diseases such as liver diseases, anaemia, cardiovascular disease, cancer, and Alzheimer’s owing to the inhibition of oxidation of low-density lipoproteins [5,6,7]. In addition, it has fruit acids and a fibrous structure that help clean the blood and regulate the functioning of the kidney and intestinal system [8]. On the other hand, biogenic amines (B.A.s) among bioactive compounds conducted on different grape varieties have been intensively investigated recently due to their effects on the quality, safety, and nutraceutical properties of fruit juices and wines [4]. Due to the fact that many authors have reported that B.A.s can have many adverse effects on human health, especially on susceptible individuals, as well as positive effects [9]. It has, indeed, been highlighted that the consumption of high concentrations of B.A.s, which are low molecular weight compounds produced by the decarboxylation of amino acids, may cause various health problems such as skin irritation, headache, blushing, itching, tachycardia, hypertension, impaired breathing, hypotension and vomit, kidney failure, anaphylactic shock [10,11,12].

Some amines such as agmatine, spermidine, serotonin, histamine, tryptamine, spermine, dopamine, norepinephrine, ethanolamine, phenylethylamine, putrescine, and tyramine in grapes have been reported by some authors [13,14]. It has, indeed, been noted that naturally occurring BAs [1,4-butanediamine = putrescine; 4-aminobutil guanidine = agmatine; 4-(2-aminoethyl) phenol = tyramine; 1,5-pentanediamine = cadaverine; 2-phenylethylamine = phenylethylamine; 4-aminomethylimidazole = histamine; (4-aminobutil) (4-[4-aminobutylamino] butyl) amine = spermine; 3-(2-aminomethyl) indole = tryptamine; N-(3-aminopropyl)-1,4-butanediamine = spermidine] are among the factors contributing to the quality of grapes and wines [15,16]. It has been detected in previous reports that these amines are both generally found in musts, and their values are affected by growing conditions, grape variety, degree of ripening, soil type, drying conditions of grape, and composition [17,18,19]. It was, indeed, highlighted that there was a relationship between varieties and B.A. content in seven different grape varieties under sterile conditions, except for agmatine [20,21]. It is well documented that the concentration and nature of B.A.s present found in wines, in general, depend mainly on the composition of the grape juice in literature by many authors [22,23,24]; however, we observed the lack of a synthesis study regarding the effect of B.A.s on quality characteristics throughout berry ripeness in grape varieties with foxy and muscat taste. As mentioned above, although there are many lines of research examining BAs in grapes and grape-derived products, there are still many possibilities to explore from microbiological, technological, analytical, and toxicological perspectives. Therefore, information on the variations or amounts of B.A.s in grapes and their derived products is mainly used as grape quality indicators and can assist in providing many insights into their potential impact on consumer health. Considering limited information on BA variation throughout berry ripeness in grape varieties with foxy and muscat taste, controlling these compounds can be of great importance to understanding the formation, reduction, and monitoring of B.A.s throughout berry ripeness, and even of the effects of B.A.s in consumers after the digestion of foods containing different levels of these compounds.

Grape varieties containing foxy (*Vitis vinifera* L. cv. Bronx Seedless) and muscat (*Vitis vinifera* L. cv. Italia) tastes are cultivated in different regions of Turkey, such as Central Anatolia Aegean, Southeastern Anatolia, and Marmara [25]. Italia is a seed variety characterized by a slight muscat flavor of fruits, a vigorous vegetative behavior, and mid-late maturity, whereas Bronx Seedless is a variety that is attracted and preferred by consumers because of its pink berries characterized by strawberry flavor. Recently, the cultivation of these new grapes that can be grown in different climatic conditions instead of traditional varieties and fresh consumption has increased in Turkey. These grape varieties have a distinct flavor from other *Vitis vinifera* grape cultivars and knowledge of the variation of BAs content in these varieties may be key for consumers. However, our knowledge of the dynamics of changes in the B.A.s profiles of Bronx Seedless and Italia table grapes throughout berry ripeness is limited. Monitoring B.A.s levels in these grape varieties can be an important marketing advantage and allow the establishment of B.A profiles for the safety and quality control of fresh consumption or by-products of these grapes. Therefore, this paper aims to present the changes in biogenic amines in Bronx Seedless and Italia table grapes throughout berry ripeness. 

## 2. Results

**Figure 1:**
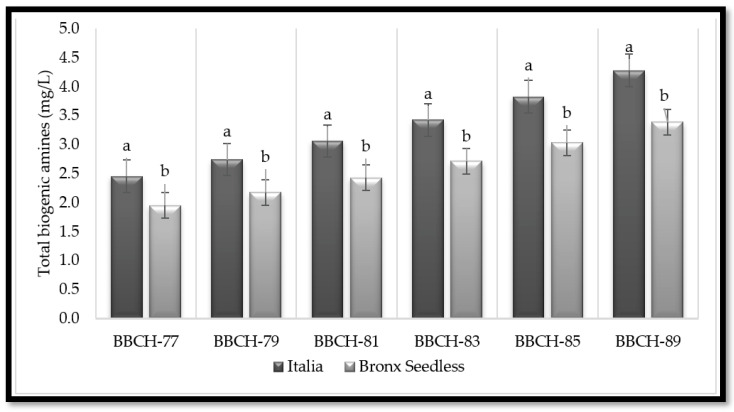
Total content of biogenic amines in Italia (black) and Bronx Seedless(grey) table grapes harvested in six different phenological stages (BBCH-77, BBCH-79, BBCH-81, BBCH-83, BBCH-85, and BBCH-89). Data are expressed as the mean of the data with their corresponding standard deviation. For a given variable and phenological stage, different letters over the bars represent significant differences (Duncan test, *p* < 0.05).

Variety significantly affected the content of B.A.s except for serotonin, and the season factor affected the content of most B.A.s. There was no significant interaction between the variety and the phenological stage. The Italia grape variety showed a higher B.A. content than the Bronx Seedless grape variety for all B.A.s analyzed, except for spermine (Spn) and serotonin (Ser). For both grape cultivars, the most common B.A.s in grape varieties was putrescine (Put), followed by histamine (His), agmatine (Agm), and tyramine (Tyr). There was wide variation in the B.A profiles, with values ranging from 0.83 (norepinephrine for Bronx Seedless) to 12.24 µg · L^−1^ (putrescine for Italia). Both putrescine (Put) and histamine (His) were mostly amines, whereas less common BAs were spermine (Spn) and norepinephrine (Nor). Generally, berries collected at BBCH-89 and BBCH-85 stages showed higher B.A. content than berries collected at other phenological stages for both grape cultivars. Put and his content reached the highest level at BBCH-89, whereas lower rates of spermine, norepinephrine, and spermidine were found at BBCH-77 when compared to BBCH-89, BBCH-85, and BBCH-83 for both grape cultivars. Considering the phenological stages, there was a linear increase in B.A.s contents of both grape varieties (Table 1). On the other hand, regarding each phenological stage, there were significant differences (*p* ≤ 0.05) between the two grape varieties. Total biogenic amine contents were higher in Italy grapes than in Bronx Seedless grapes (Figure 1).

**Figure 2:**
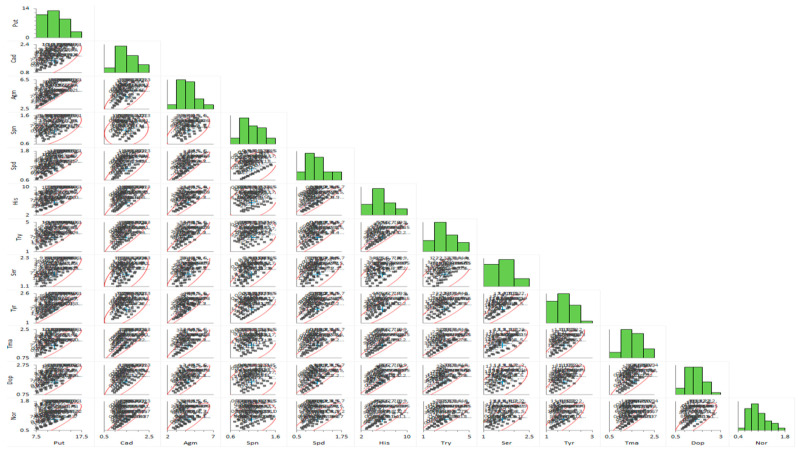
Grape varieties data correlation analysis. Scatterplot matrix representation for the entire data set belonging to biogenic amines values of grape varieties.

**Figure 3:**
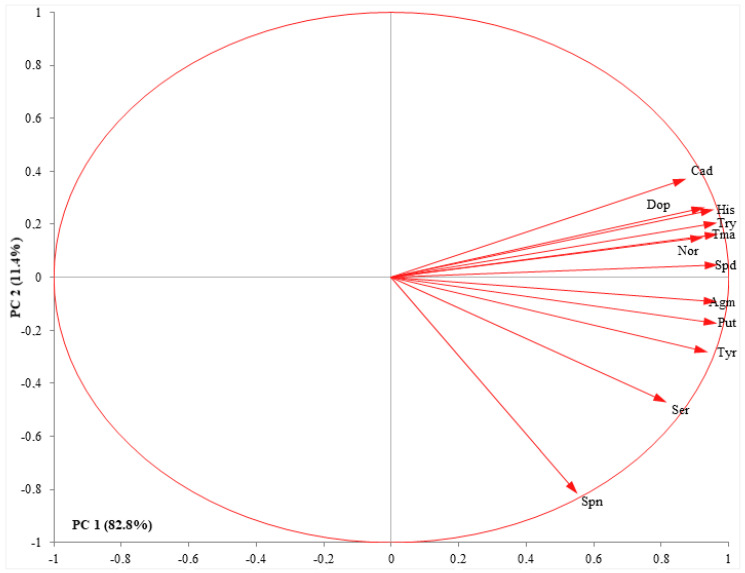
P.C.A. biplot (score and loadings plots) of berries colored by varieties. All biogenic amines are displayed. The size of the arrows indicates the contribution strength of the compound. Each point is the average of quaternaryplicate of each organic acid.

Pearson correlation analyses for the biogenic amine contents (i.e., Agm, Spd, Ser, His, Try, Dop, Nor, Cad, Tma, Put, Tyr) appear in Figure 2 for all data sets. Findings showed a positive correlation between all B.A.s of the two grape varieties. There was also a positive but strong correlation between grape varieties with B.A.s, except for Spn, Dop, His, Agm, Tyr, and Nor. Put, and Agm showed a strong correlation with other B.A.s (*p* ≤ 0.01), whereas there was no correlation between Spn and Cad (Table 2). Apart from this, a P.C.A. was performed on all of the B.A.s identified in both grape varieties to detect individual B.A. associated with the grapes (Figure 3). PC1 (reveals 82.8% of the total data) was related to the B.A.s contents, such as Agm, Spd, Ser, His, Try, Dop, Nor, Cad, Tma, Put, Tyr. PC2 (which reveals 11.4% of total data) was related to B.A.s contents (Figure 3).

## 3. Discussion

Although there have been new approaches and reports on a wide range of functions of B.A.s in wine and table grapes in the last few decades [4,22,26], to our knowledge, information on the reactions of B.A. both in grape varieties containing foxy and muscat taste and in berry ripeness stages has yet to be revealed. Previous results on the functions of B.A.s in wine and table grapes have allowed us to understand positive and negative effects on human health in greater and greater detail; however, the picture’s complexity has dramatically increased, and negative effects on human health appear in greater and greater detail; however, the complexity of the picture has greatly increased. Much previous research has focused on the effects of different applications on grape and wine chemical composition in various grape varieties [4,22,26]; however, less study has been conducted with B.A.s of grape varieties containing foxy and muscat taste. In this context, the results of our study are similar to the previous reports by researchers who analyzed the B.A.s of wine, and pomace from different varieties grown in various viticultural regions of the world. In our study, Put, His, Agm, and Try were the most abundant amino acids in raisin varieties, confirming previous reports that these B.A.s occur at high concentrations in grapes [27]. However, when the general literature is examined, the effect of grape evaluation forms or grape by-products (i.e., grape seeds, skins, wines, pomace, bagasse, stalks) on the concentration of B.A.s of raisin is not clear and consistent among the different varieties and even for the seeded and seedless grape varieties. 

Experimental observations on highly complex systems for B.A.s in grape varieties are mainly based on measurements performed in wine and must be rationalized in terms of elementary article science. The present study is, in this context, the first report of a synthesis study regarding the effect of B.A.s on quality characteristics throughout berry ripeness in grape varieties containing foxy and muscat tastes. Based on our results, we can state that there was wide variation in the total B.A.s between grape varieties, with values ranging from 12.24 (Put) to 0.83 µg · L^−1^) (Nor). In addition, Put, His, Agm, and Try were the majority amine, coinciding with the results of Gomez et al. [27]. Moreover, some authors who agree with our findings reported on the presence of primary amines being generally histamine, tyramine, putrescine, cadaverine, tryptamine, agmatine, phenylethylamine, methylamine, isoamylamine, and ethylamine in wine products obtained from different grape [10,28]. 

On the other hand, the Bronx Seedless grape variety showed lower content of all B.A.s than the Italia grape variety, except for Spn and Spd (Table 1). This was not surprising given the reports that the content of B.A. in grapes varies by variety [24,29]. The Spn and Spd contents were relatively low for both varieties, and this could be explained by the fact that probably by the metabolism of Agm, which is the most abundant amino acid in grape or Spn and Spd originate from Put, the latter being produced by decarboxylation of ornithine [30]. Although Put has also been found that this amine may be present in berries without external microbial contamination, it has been related to poor sanitary conditions of grapes [28]. Some studies have highlighted that the quantity of amines in food depends not only on the amount of microorganisms present but on the activity of the decarboxylase enzyme on specific amino acids and the favourability of the enzymatic conditions, like, pH, temperature [28,29,30].

As far as phenological stages are concerned, as the berry development progresses, individual B.A.s concentrations also rise, and there was a linear increase in all B.A.s contents of both grape varieties. The berries of BBCH-89 and BBCH-85 stages reached higher B.A. content than the berries of other phenological stages. The most common B.A.s in grape varieties were Put, His, Agm, and Try, whereas among these amines, Spn, Nor, and Spd were the lowest (Table 1). It has been reported that some bio-components exhibit patterns of decline and subsequent accumulation during ripening, suggesting their covalent association with other cellular compounds or utilization and degradation for the biosynthesis of other compounds [31]. However, there is not much information in the literature about the anabolism or the catabolism of B.A.s during berry development and ripening. Logically here, we hypothesize that the smaller berry size in very early berry development contains low concentrations of B.A. compared to larger berry size. On the other hand, we think that B.A.s could have increased in musts of berry due to the hydrolysis of hydroxycinnamic amide compounds throughout berry ripeness. It is, indeed, known that amino acid precursors could accumulate in berries as well during berry ripeness, supporting a further B.A.s increase. It has also been noted that amino acids vary depending on the berry development stages [32], which confirms our hypothesis. Although it is known that there is no consensus on the correlation between amino acids in the environment and total B.A.s [21], this point should not be neglected by researchers.

Regarding the Pearson correlation values calculated to determine the relationships between individual B.A.s analyzed in all data, a significant positive correlation (*p* ≤ 0.05) was obtained among B.A.s (Figure 2). This is consistent with the results that there is a significant correlation between some B.A.s identified in raisins [14]. Most significant correlations for data obtained from both grape varieties show a high confidence level (*p* ≤ 0.05), except for His, Dop, Tyr, Spn, Agm, and Nor (Table 2). A marked correlation was determined between Put and other B.A.s (*r* values greater than 0.73, with *p* ≤ 0.05); between Cad and other B.A.s, except for Spn, Ser, and Tyr (r values greater than 0.61, with *p* ≤ 0.05). The strongest correlation was found among Dop, Tma, Nor, Try, and His amines, whereas the weakest correlation was found between Spn and Cad (Table 2). It has been noted that there is a high correlation between serotonin, spermidine, spermine, and tryptophan [4], which is consistent with our results. In addition, the relationship among cadaverine, tryptamine, and phenylethylamine [33], between histamine-tyramine [34], putrescine-tyramine [35], or some amines [36] have been reported in different works for wines. On the other hand, we opted to compare the detected findings by P.C.A. in terms of creating a descriptive model for grouping B.A.s as a function of grape varieties throughout berry ripeness. A PCA was obtained that detected for (PC1 + PC2) 94.2 % of the variance in the twelve B.A.s for grape varieties in our findings. Cad, Dop, His, Try, Tma, Nor, and Spd were close to each other in the second quadrant, whereas the Agm, Put, Try, and Ser was close to each other (except for Spn) and was located in the third quadrant (Figure 3). This correlation among B.A.s concentration during berry ripening in our study supports the results of Ates et al. [14], who found a high correlation between spermine, tryptamine, putrescine, agmatine, and dopamine.

Given the dual importance (quality and health effects) of B.A.s, on the other hand, efforts must be made to control B.A.s in grape products and regulatory limits for B.A.s have not yet been established by the OIV (Organization International de la Vigne et du Vin) for fresh grape, raisins, and wines; however, Lehtonen [37], stated that for France 8 mg/L, Belgium 5–6 mg/L, Austria 10 mg/L, Holland 3.5 mg/LGermany 2 mg/L, and Switzerland 10 mg/L as the maximum limit for histamine in wine are recommended. A legal limit for histamine in wine does not exist in either the European Union or Austria [37]. Although our findings for histamine are lower than the above-mentioned limit values, the maximum BA limit that is generally considered safe for consumers is not yet known for fresh grapes. It has been reported that histamine and tyramine are generally assumed to be the most toxic amines among B.A.s European Food Safety Authority [38], (2011), but to the best of our knowledge, there is no scientific data to confirm this yet. Despite the increase in B.A.s content as the berry development progresses in our study, these values are well below the values considered harmful to human health and do not cause any problems related to berry quality.

## 4. Materials and Methods

### 4.1. Plant Material and Sample Preparation

The study was conducted in 2021 on twenty-year-old Bronx Seedless (New York 8536 × Sultanina) and Italia (Bicane × muscat Hamburg) vines grafted onto 5 B.B. root-stock in the Manisa Province (Manisa Viticulture Research Institute), Aegean Region, Turkey (27°23′57.36″ East Longitude and 38°37′57.14″ North Latitude at 3.3 m above sea level. Bronx Seedless and Italia vines were planted at 2.0 m between vines and 3.0 m between rows. Vines have a high trunk (about 1 m) cordon trellis system with 12–15 shoots per vine and one cluster per shoot at a northwest orientation, and vines were spur-pruned. Healthy berries (450 per cultivar) were randomly harvested in triplicate from the cluster’s bottom, middle, and top parts. Samplings were carried out on July 27 (the first week before veraison; stage BBCH-77), and the latest sampling was August 28 (harvest time; BBCH-89), for a total of six selections. Based on BBCH scale published by Lorenz et al. [39], clusters were harvested in six different stages as follows: BBCH-79, BBCH-81, BBCH-83, BBCH-85 (these stages follow in order; begin berry touch, berry touch complete, berries begin to brighten in color, berries brightening in color, softening of berries, berries ripe for harvest). Clusters were collected, put into plastic bags, and transported at 4 °C to the laboratory, where they were stored at −80 °C until analysis.

### 4.2. Chemicals

Standard solutions of spermine (in the 1 to 30 mg/L range), agmatine, spermidine, serotonin, histamine, tryptamine, dopamine, norepinephrine, cadaverine, trimethylamine, putrescine, and tyramine were obtained from Sigma-Aldrich Chemie, Steinheim, Germany. 

### 4.3. Isolation of Amines from Grape Varieties

Berries (5 g) were homogenized using an Ultra-turax homogenizer, including 0.5 mL of 70% perchloric acid. Homogenate (X g) was centrifuged at 10,000 rpm for 10 min. The supernatant was recovered, filtered over a 0.22 mm membrane, and diluted with 10% perchloric acid to the initial homogenate weight. The sample was then filtered over 0.45 µm and injected into the HPLC.

### 4.4. Identification of Amines from Grape Varieties by HPLC

Biogenic amines (B.A.) were separated and quantified according to the method of Nagy et al. [40], with modifications. Samples were injected on a reverse phase column (Bondapak C18, 300 × 3.9 mm, 10 mm; Waters, Milford, MA, USA) mounted on a Waters Alliance Liquid Chromatograph attached to a Waters 474 fluorescence detector (Milford, MA, USA). Post-column derivatization (2-mercaptoethanol, o-phtalaldehyde) was used to improve detection. Peaks were identified using authentic standards. Calibration curves in the range of 1 to 30 mg/L (spermine) and 0.1 to 10 mg/L (agmatine, spermidine, serotonin, histamine, tryptamine, dopamine, norepinephrine, cadaverine, trimethylamine, putrescine, and tyramine) were used for quantitation. The biogenic amine content of samples was expressed in µg · L^−1^ fresh weight.

### 4.5. Statistical Analysis 

Descriptive statistics for the studied variables were analyzed considering a completely randomized design with the factorial arrangement, accounting for two grape varieties involving six phenological stages. The variables were subjected to a variety analysis (ANOVA) that was performed using SPSS 21.0 (SPSS Inc., Chicago, IL, USA). Duncan’s test detected the significance of the differences in data (*p* ≤ 0.05). The Pearson correlation coefficients were evaluated as scatterplot matrices using Analyse-it statistical software, and they were significant at the 0.01 (**) and 0.05 (*) levels.

## 5. Conclusions

Although scientific advances have been achieved in understanding the biochemical, molecular, and physiological aspects of berry ripening in grapes, there is no previous research on the change of B.A.s throughout berry ripeness. Therefore, this study is the first report on B.A.s. The findings showed that Bronx Seedless table grapes presented higher contents of Put, Cad, Agm, Spd, His, Try, Ser, Tyr, Tma, Dop, Nor, and lower contents of Spn than Italia table grapes. The analyzed B.A.s concentration increased linearly as the berry ripened, and there reached the highest level in BBCH-77 and BBCH-89 stages. In addition, a significant positive correlation was detected between B.A.s and a strong correlation between Dop, Tma, Nor, Try, and His amines. Given the therapeutic value and importance of some amines of these grapes for humans, the pharmaceutical industry may capitalize on the potential human health benefits of pharmaceutical preparations for plants, animals, and humans. Indeed, it is likely that soon, we will be able to see the processing of pre-and post-harvest food crops and the production of fruits and vegetables with higher amine levels by combining traditional and modern growing approaches.

## Figures and Tables

**Table 1:** Biogenic amines content (µg · L^−1^) of Italia and Bronx Seedless table grapes harvested in six different phenological stages (BBCH-77, BBCH-79, BBCH-81, BBCH-83, BBCH-85, and BBCH-89).

Variety (V)	Put	Cad	Agm	Spn	Spd	His	Try	Ser	Tyr	Tma	Dop	Nor
Italia	12.24 ± 0.12 a	1.76 ± 0.03 a	4.62 ± 0.26 a	0.99 ± 0.08 b	1.23 ± 0.12 a	6.90 ± 1.26 a	3.45 ± 1.18 a	1.64 ± 0.68 a	1.84 ± 0.87 a	1.78 ± 0.43 a	1.93 ± 0.52 a	1.15 ± 0.12 a
Bronx Seedless	10.71 ± 0.56 b	1.21 ± 0.48 b	3.87 ± 0.26 b	1.16 ± 0.62 a	0.92 ± 0.08 b	4.47 ± 0.23 b	2.33 ± 0.15 b	1.59 ± 0.19 a	1.66 ± 0.05 b	1.30 ± 0.08 b	1.30 ± 0.11 b	0.83 ± 0.06 b
Phenological stage (S)												
BBCH-77	8.66 ± 1.13 e	1.22 ± 0.06 b	3.17 ± 1.01 e	0.82 ± 0.01 e	0.79 ± 0.05 d	4.08 ± 0.21 d	2.02 ± 0.10 d	1.22 ± 0.13 d	1.30 ± 0.18 f	1.16 ± 0.23 c	1.22 ± 0.10 c	0.73 ± 0.11 d
BBCH-79	9.62 ± 1.18 de	1.31 ± 0.11 b	3.54 ± 0.16 de	0.91 ± 0.02 dc	0.89 ± 0.07 d	4.61 ± 0.18 cd	2.31 ± 1.03 cd	1.36 ± 0.03 cd	1.46 ± 0.26 e	1.29 ± 0.16 cd	1.36 ± 0.18 c	0.82 ± 0.16 cd
BBCH-81	10.70 ± 0.23 cd	1.42 ± 0.62 ab	3.95 ± 0.18 cd	1.01 ± 016 cd	1.00 ± 0.05 cd	5.22 ± 1.12 bcd	2.63 ± 0.62 bcd	1.51 ± 0.05 c	1.63 ± 0.07 d	1.44 ± 0.19 bcd	1.51 ± 0.12 bc	0.92 ± 0.08 bcd
BBCH-83	11.90 ± 2.08 c	1.53 ± 0.03 ab	4.41 ± 0.09 bc	1.12 ± 0.09 bc	1.12 ± 0.06 bc	5.91 ± 1.07 abc	3.01 ± 0.15 bc	1.68 ± 0.28 c	1.82 ± 0.19 c	1.60 ± 0.25 bc	1.68 ± 0.08 abc	1.03 ± 0.01 abc
BBCH-85	13.23 ± 2.16 b	1.66 ± 1.02 ab	4.92 ± 0.83 ab	1.24 ± 0.02 b	1.26 ± 0.24 ab	6.69 ± 1.09 ab	3.44 ± 0.98 ab	1.87 ± 0.62 b	2.04 ± 0.69 b	1.78 ± 0.41 ab	1.87 ± 0.65 ab	1.16 ± 0.16 ab
BBCH-89	14.72 ± 2.61 a	1.79 ± 0.066 a	5.49 ± 0.98 a	1.38 ± 0.07 a	1.41 ± 0.31 a	7.57 ± 2.16 a	3.92 ± 0.86 a	2.07 ± 0.75 a	2.28 ± 0.53 a	1.98 ± 0.26 a	2.07 ± 0.45 a	1.30 ± 0.29 a
Significance												
V	0.0000	0.0000	0.0000	0.0000	0.0000	0.0000	0.0000	0.0000	0.0000	0.2724	0.0000	0.0000
S	0.0000	0.0000	0.0000	0.0000	0.0000	0.0000	0.0000	0.0000	0.0000	0.0000	0.0000	0.0000
V × S	0.8800	0.9291	0.8272	0.7403	0.0813	0.0738	0.4716	0.9992	0.9091	0.7115	0.3302	0.8841

Data are expressed as mean of the data. aSignificance (*p*-value) of variety (V), season (S), and V–S interactions. For a given factor and significance (*p* < 0.05), different letters within a column represent significant differences (Duncan test, *p* < 0.05). Put; Putrescine, Cad; Cadaverine, Agm; Agmatine, Spn; Spermine, Spd; Spermidine, His; Histamine, Try; Tryptamine, Ser; Serotonin, Tyr; Tyramine, Tma; Trimethylamine, Dop; Dopamine, Nor; Norepinephrine.

**Table 2:** Pearson correlation between biogenic amines in grape varieties. Significant correlations are reported for 0.01 < *p* < 0.05 (**) and *p* < 0.01 (*). The color intensity is proportioned to the Pearson Index.

Pearson’s r	Put	Cad	Agm	Spn	Spd	His	Try	Ser	Tyr	Tma	Dop	Nor
Put	1											
Cad	0.739 **	1										
Agm	0.984 **	0.757 **	1									
Spn	0.684 **	0.193 ns	0.593 **	1								
Spd	0.948 **	0.814 **	0.958 **	0.487 **	1							
His	0.891 **	0.897 **	0.928 **	0.302 *	0.952 **	1						
Try	0.912 **	0.915 **	0.908 **	0.390 **	0.933 **	0.968 **	1					
Ser	0.809 **	0.613 **	0.776 **	0.819 **	0.744 **	0.642 **	0.666 **	1				
Tyr	0.963 **	0.689 **	0.971 **	0.721 **	0.902 **	0.851 **	0.832 **	0.886 **	1			
Tma	0.891 **	0.961 **	0.895 **	0.416 **	0.922 **	0.943 **	0.967 **	0.740 **	0.842 **	1		
Dop	0.889 **	0.838 **	0.910 **	0.295 *	0.966 **	0.975 **	0.956 **	0.577 **	0.806 **	0.905 **	1	
Nor	0.818 **	0.952 **	0.811 **	0.400 **	0.841 **	0.895 **	0.933 **	0.773 **	0.798 **	0.955 **	0.827 **	1

**. Correlation is significant at the 0.01 level; *. Correlation is significant at the 0.05 level; ns; not significant.

## Data Availability

Not applicable.
